# Strengthening primary health care through primary care and public health collaboration: the influence of intrapersonal and interpersonal factors

**DOI:** 10.1017/S1463423617000895

**Published:** 2018-04-12

**Authors:** Ruta K. Valaitis, Linda O’Mara, Sabrina T. Wong, Marjorie MacDonald, Nancy Murray, Ruth Martin-Misener, Donna Meagher-Stewart

**Affiliations:** 1 Associate Professor and Dorothy C. Hall Chair in Primary Health Care Nursing, School of Nursing, Faculty of Health Sciences, McMaster University, Hamilton ON, Canada; 2 Associate Professor, School of Nursing, McMaster University, Hamilton, ON, Canada; 3 Professor, School of Nursing, University of British Columbia, Vancouver, BC, Canada; 4 Professor, School of Nursing, University of Victoria, Victoria, BC, Canada; 5 Research Coordinator, School of Nursing, McMaster University, Hamilton, ON, Canada; 6 Professor, School of Nursing, Dalhousie University, Halifax, NS, Canada; 7 Associate Professor, School of Nursing, Dalhousie University, Halifax, NS, Canada

**Keywords:** collaboration, partnership, primary care, primary health care, public health, relational practice

## Abstract

**Aim:**

The aim of this paper is to examine Canadian key informants’ perceptions of intrapersonal (within an individual) and interpersonal (among individuals) factors that influence successful primary care and public health collaboration.

**Background:**

Primary health care systems can be strengthened by building stronger collaborations between primary care and public health. Although there is literature that explores interpersonal factors that can influence successful inter-organizational collaborations, a few of them have specifically explored primary care and public health collaboration. Furthermore, no papers were found that considered factors at the intrapersonal level. This paper aims to explore these gaps in a Canadian context.

**Methods:**

This interpretative descriptive study involved key informants (service providers, managers, directors, and policy makers) who participated in one h telephone interviews to explore their perceptions of influences on successful primary care and public health collaboration. Transcripts were analyzed using NVivo 9.

**Findings:**

A total of 74 participants [from the provinces of British Columbia (*n*=20); Ontario (*n*=19); Nova Scotia (*n*=21), and representatives from other provinces or national organizations (*n*=14)] participated. Five interpersonal factors were found that influenced public health and primary care collaborations including: (1) trusting and inclusive relationships; (2) shared values, beliefs and attitudes; (3) role clarity; (4) effective communication; and (5) decision processes. There were two influencing factors found at the intrapersonal level: (1) personal qualities, skills and knowledge; and (2) personal values, beliefs, and attitudes. A few differences were found across the three core provinces involved. There were several complex interactions identified among all inter and intra personal influencing factors: One key factor – effective communication – interacted with all of them. Results support and extend our understanding of what influences successful primary care and public health collaboration at these levels and are important considerations in building and sustaining primary care and public health collaborations.

## Background

Primary health care systems can be strengthened by building stronger collaborations between primary care (PC) and public health (PH). Stronger collaborations can lead to more integrated systems, universal coverage, improved access to care, and ultimately improved health outcomes (Institute of Medicine (IOM), 2012; Martin-Misener *et al*., 2012; Sutton and Long, 2014; Castrucci and Sprague, 2015; Valentijn *et al*., 2015; Booth *et al*., 2016). An international literature review of PC and PH collaboration reports that collaboration is an important way to achieve the principles of equity and access to health care (Specchia *et al*., 2013). In Canada, there are examples of PC and PH collaborations that have addressed the following areas: infection control (Hogg *et al.*, 2006); chronic disease prevention, targeted prevention and care for child health (Stevenson Rowan *et al*., 2007); cancer screening, pre and postnatal care, and sexual health (de Guzman, 2010); as well as mental health and addictions management (Anderson and Larke, 2009). Importantly, collaboration cannot be developed or strengthened without successful relationship practice interactions, many of which happen at the interpersonal level (Holmes and Marra, 2004; Ness, 2009). Although there is literature that explores interpersonal factors (between individuals) that can influence successful inter-organizational collaborations, a few papers have specifically explored PC and PH collaboration. Furthermore, no papers were found that considered intrapersonal level (within an individual) influencing factors. This paper addresses these gaps by exploring interpersonal and intrapersonal factors influencing PC and PH collaboration in the Canadian context.

This study builds on a scoping literature review of PC and PH collaboration that found negative attitudes and a lack of shared team values and beliefs were barriers to collaboration (Martin-Misener *et al*., 2012), whereas valuing all team members and developing and maintaining good relationships, trust, and respect were facilitators. Effective communication, decision-making strategies, regular staff meetings, and attention to process at the interpersonal level also promoted collaboration.

San Martín-Rodríguez *et al*. identified components of successful relationship-based interactions in inter-organizational collaborations, although they were not specific to PC and PH. These included: willingness to collaborate, trust, communication, and mutual respect. Willingness to collaborate is based on group cohesion, which, in turn, is based on professional constancy in the group, and education, previous experience, and personal maturity. Trust, a key element, requires time, effort, and patience to develop, is based on confidence in one’s own role and that of others, and is dependent on competence, skills, knowledge, and experience. Trust between professionals was also identified by D’Amour *et al*. (2008: 5) as an indicator of developing interorganizational collaborations wherein ‘collaboration is possible only when [professionals] have trust in each other’s competencies and ability to assume responsibilities (that is, when goodwill exists).’ Mutual aquaintanceship was another indicator that was explained as professionals knowing each other personally and professionally ‘if they are to develop a sense of belonging to a group and succeed in setting common objectives’ (D’Amour *et al*., 2008: 5). Communication is crucial for relationship development (Doane and Varcoe, 2007). Constructive negotiation among professionals in collaborations requires that workers understand how their work contributes to team outcomes and how to communicate the content of their contributions.

It has been argued that strong relational practice between interprofessional teams is vital to ensure the delivery of integrated services that are patient, family, and community-centered (Schwind *et al*., 2016). Ultimately, it can positively impact client outcomes (D’Amour *et al*., 2005). D’Amour *et al*. (2005: 117) further argue that we have a limited understanding of the complex relationships between professionals who have been socialized to work within a ‘discipline-based vision’ that builds competition rather than collaboration. This threatens the ability to build and sustain integrated intersectoral collaborations that are needed for a strong primary health care system (World Health Organization, 2003). Therefore, gaining a stronger understanding of what is needed to build strong relational practice among professionals will be useful to managers and practitioners implementing collaborative practice initiatives. The objectives of this paper are to: (1) identify interpersonal and intrapersonal level factors influencing PH and PC collaboration, and (2) examine the relationships between these factors. Table 1 provides definitions of PH, PC and collaboration.

**Table 1:** Definition of terms

Primary care ‘…the first point of entry to a health care system, the provider of person-focused care (not disease oriented) over time for all but the most uncommon conditions and the part of the system that integrates or co-ordinates care provided elsewhere or by others.’ (Starfield, 1998)
Public health ‘to promote, protect and improve, and when necessary, restore the health of individuals, specified groups, or the entire population. It is a combination of sciences, skills, and values that function through collective societal activities and involve programs, services, and institutions aimed at protecting and improving the health of all people.’ (Public Health Agency of Canada, 2008: 13)
Collaboration ‘a recognized relationship among different sectors or groups, which have been formed to take action on an issue in a way that is more effective or sustainable than might be achieved by [any one group or sector] acting alone.’ (Public Health Agency of Canada, 2008: 9)

## Methods

In this paper we report on a nested study that was part of a larger program of research examining PC and PH collaborations in Canada (Valaitis *et al*., 2012). The larger study was loosely guided by San Martín-Rodríguez *et al*.’s framework (2005) that identified three collaboration determinants: systemic (outside the organization); organizational (in the environment where the collaborations take place); and interactional determinants (interpersonal interactions between team members). Given the complexity of collaboration, this nested study reports in depth on interactional determinants, which we refer to as interpersonal and intrapersonal influencing factors of PH and PC collaboration. Forthcoming papers will explore: influencing factors at the organizational and systemic levels; the nature of PC and PH collaboration that focuses on actors, settings, motivations, the structure and activities of PC and PH collaborations in the Canadian context (the core) and; relationships across all levels and the core to form a comprehensive ecological framework for successful PC and PH collaboration.

This interpretive descriptive qualitative study (Thorne, 2008) involved interviews with key informants who were eligible if they: (a) provided direct care in either PH or PC; (b) had responsibility for how services in PC or PH were organized or delivered; or (c) had knowledge about or experience in PC and PH collaboration. We used stratified purposive sampling and a snowball technique (Patton, 1990) to recruit an approximately equal number of participants from each sector and participating province [British Columbia (BC), Ontario (ON), and Nova Scotia (NS)], and from national PH and PC organizations or known collaborations in other Canadian provinces. We recruited policy makers, directors, managers, and direct service providers with diverse disciplines. We conducted interviews from over 30 participants from each sector (others worked in both PC and PH) to capture diverse views.

Our multi-disciplinary research team and program advisory committee provided names of key informants. Participants were contacted by email with a follow-up call to explain the study and arrange a convenient interview time. We asked these key informants to identify other potential participants who met our eligibility criteria. After faxing/emailing a signed consent, in-depth interviews were conducted by phone or in person at a mutually convenient location. Participants were asked open-ended questions about their perceptions of factors that influenced building and maintaining PH and PC collaborations. Interviews lasted between 45 and 90 min, were audio-taped, transcribed, and anonymized. Procedures were approved by 10 university or regional health authority research ethics boards.

Interview data were analyzed using interpretive thematic analysis, an approach that borrows from established qualitative analytic techniques, such as the constant comparative method of grounded theory (Thorne, 2008: 151). Conventional grounded theory approaches to analysis have been used as pragmatic ‘tools’ in interpretive qualitative inquiry when the aim is to explore and uncover commonalities and patterns and understand social phenomena (Strauss and Corbin, 1998; Thorne, 2008). Although the research was not directed toward the development of a grounded theory, we used constant comparative analysis in which each incident was compared with other incidents, incidents were compared with identified concepts or themes, and each concept was compared with other concepts or themes. Data were organized into codes using NVivo 9 (Richards, 1999). Authors and research assistants coded three transcripts independently. To capture as much variation as possible in responses, transcripts were selected to include a key informant from each province and sector as well as different disciplines. After first-level coding (Miles and Huberman, 1994) was conducted the team categorized these codes into second-level codes or pattern codes and generated a draft code book. The coding scheme was further refined by having team leads in each province independently code another subset of transcripts. Constant comparison (Strauss and Corbin, 1998) was applied and the coding structure was finalized over multiple team meetings. Credibility of analysis was continually evaluated with the full team including experts in both qualitative research and PC and PH. We conducted matrix queries in NVivo 9 to examine potential cross-sectoral, cross-provincial differences in perceptions and interactions among influencing factors. Data displays containing numbers of respondents from each province or sector for each factor were created with matrix queries using the Boolean operator – ‘AND.’ Sandelowski (2001) argues that displaying data numerically helps to see patterns more clearly, which can generate new questions or help to understand meaning and sharpen the focus of results. We also conducted queries to identify potential relationships among factors with matrix queries using the search criterion ‘NEAR content.’ We explored text passages that were coded for one influencing factor and were located ‘near’ text coded for another factor. These were manually reviewed to identify potential relationships.

## Findings

### Demographics

Seventy qualitative interviews were conducted with 74 participants [(BC, *n*=20, 27.0%) (ON, *n*=19, 25.7%) (NS, *n*=21, 28.4%) (National, *n*=14, 18.9%)]. At participants’ request, two interviews were done in a group, one with two informants and the other three. Participants were employed in or responsible for PC (*n*=32; 43.2%), PH (*n*=31; 41.9%), both sectors (*n*=8; 10.8%) or neither sector (eg, researchers) (*n*=3; 4.1%). Roles and disciplines of participants are shown in Table 2. Participants had 5–40 years of healthcare experience, with 68% having over 20 years. Most participants were female (*n*=58; 78.4%).

**Table 2:** Roles and disciplines of participants

	Frequency	%
Role		
Direct service providers	17	22.9
Senior program managers	14	18.9
Executive officers	11	14.9
Middle managers	10	13.5
Policy makers	8	10.9
Other (eg, health educator, coordinator, consultant, researcher)	14	18.9
Total	74	100
Discipline		
Physicians	14	18.9
Registered nurses (not including public health nurses)	14	18.9
Public health nurses	11	14.9
Business administrators	8	10.8
Nurse practitioners	7	9.5
Other professional disciplines (health promoter, dietitian, social worker, epidemiologist, psychologist, public health dentist, etc.)	20	27.0
Total	74	100

### Inter and intrapersonal interactional factors

Each factor that emerged from our data and their respective elements (presented in italics) are summarized in Table 3. Five influencing factors were identified at the interpersonal level: (1) Trusting and Inclusive Relationships; (2) Shared Values, Beliefs, and Attitudes; (3) Role Clarity; 4) Effective Communication; and (5) Effective Decision Processes. Two factors were at the intrapersonal level: (1) Personal Qualities, Knowledge, and Skills; and (2) Personal Values, Beliefs, and Attitudes. We describe each influencing factor and present differences between provinces where they exist. Following the presentation of each factor and its elements, a brief discussion follows in relation to the existing literature. Similarly, relationships among factors at inter and intrapersonal levels are presented with a brief discussion. The paper concludes with an overall discussion and conclusions.

**Table 3:** Interactional factors affecting collaboration

	Elements
Interpersonal level factors	
Trusting and inclusive relationships	Positive relationship development and maintenance Collaborative working styles Trust and respect of others
Shared values beliefs and attitudes	Openness and belief in collaboration Values, attitudes, philosophies related to change
Role clarity	Understanding/agreement of roles and mandates Flexible roles/adaptability
Effective communication	Exchange of information Facilitated, engaged dialogue
Effective decision processes	Practitioner problem solving Practitioner decision making
Intrapersonal level factors	
Personal knowledge, qualities, and skills	Experience/knowledge in collaboration Leadership skills in collaboration Practitioner personal characteristics
Personal values, beliefs, and attitudes	Willingness to collaborate Responsiveness to patient, community and provider needs

### Interpersonal factors

#### Trusting and Inclusive Relationships

The influencing factor – Trusting and Inclusive Relationships – incorporates the following elements: (a) *positive relationship development and maintenance*, (b) *collaborative working styles*, and (c) *trust and respect of others*.

Positive relationship development and maintenance involved the process of relationship building to foster sustainable collaboration. A PC nurse practitioner explained, ‘I felt it was really important to form a connection with each public health nurse (PHN)’ [BC-08]. In explaining essential components of collaboration, she added, ‘there’s probably only one, it’s relationship’ [BC-08]. Positive relationship development takes time. A PC social worker explained, ‘…the benefit of being in a position for a period of time, as I have been here, is you develop these on-going relationships with [individuals] at PH…’ [ON-03] Having a shared work history, even working within another sector, enabled positive relationship development and maintenance. A PC nurse explained, ‘… by doing the liaison [role] at the hospital for a number of years, some of [our] relationships had been established’ [NS-15]. A collaborative working style also influenced successful collaboration. One example was using ‘a non-hierarchical network’ [BC-14] approach which neutralized power among players. Face-to-face meetings demonstrated a collaborative working style. A nurse practitioner proactively ‘made a point of going and visiting [the PH] office and meeting the individuals. So there was a face with the name when I called’ [NS-20].

The element – trust and respect of others – and its significance in building relationships was described by a PC nurse practitioner: ‘You want to be professional. You want to show that you’re supporting whatever they are doing and that you have something to offer them, right? And that’s how you kind of build the trust and build loyalty’ [BC-10]. On the other hand, a lack of respect and ‘power over’ relationships undermined trust. As described by a PH professional:

Collaboration isn’t something that we’re born to do. We’re born to be competitive […]. It all comes down to mutual respect and mutual trust. And if you’re never exposed to the other people, except as someone who’s your gopher, then how do you build respect and trust?

[BC-06]

Kempe *et al*. (2014) argue that the development of personal connections was essential to lay the groundwork in a PC and PH collaboration to improve immunization rates. Others studying inter-organizational collaborations have found that this strategy can help build trusting and inclusive relationships (Walker *et al*., 2009). A study of networks of public and not-for-profit organizations showed that frequent interactions can help build trust (Lambright *et al*., 2010). When staff turnover occurs, it is important to invest time in new personnel to teach them about collaboration and building trusting relationships (Sloane *et al*., 2009). This highlights the notion that building trust in inter-organizational collaborations is a cyclical process that requires constant attention (Vangen and Huxham, 2003).

#### Shared Values, Beliefs, and Attitudes

Two elements describe the influencing factor shared values, beliefs and attitudes: (a) *openness and belief in PC and PH collaboration* and (b) *values, attitudes, and philosophies related to change*. The element openness and belief in PC and PH collaboration was enabled when people valued collaboration, shared interests, and had common goals. A national representative with PC and PH experience stated, ‘…you just have to be open to change and realize that you’re here for the client and the best that you can give your client is the best outcome for them’ [Nat-11]. Skepticism about the benefits of collaboration between PH and PC and among disciplines was an impediment to collaboration.

Values, attitudes and philosophies related to change is another element describing this factor. For example, resistance to change was viewed as a barrier to PC and PH collaboration, while attitudes that were open to change were enablers. A PHN explained: ‘I think it’s that if people come in with that territoriality or arrogance or whatever you want to call it, that’s when it won’t work. I know I’ve sounded positive about the family physician [but] it wasn’t positive at the beginning’ [ON-16].

The quote above suggests the presence of unspoken power relations between professions (eg, physicians and nurses) and PH and PC sectors. There has been a long legacy in Canada of physicians’ autonomy and self-management (Hutchison *et al*., 2011), which may influence physicians’ openness to change. Research has shown that interprofessional education in primary care has resulted in positive impacts on attitudes to collaboration as well as belief in the benefit of collaboration (Robben *et al*., 2012) and may be one strategy to overcome such barriers among disciplines and sectors.

#### Role Clarity

Role Clarity is composed of two elements: (a) *understanding of and agreement about roles and mandates* and (b) *flexibility and adaptability*. Understanding of and agreement about roles and mandates relates to discipline-specific and sector-mandated day-to-day activities that were misunderstood by individuals. For example, a nurse said: ‘A lot of PC providers don’t understand what PH does in general, other than baby immunizations and new mum visits, […] I don’t know that they always have a really great sense of what our role is’ [BC-10]. Who does what needs to be clear as exemplified by this PC physician’s concern: ‘I think the understanding between PC physicians and PH about who tells what to whom [with respect to HIV reporting] and where the information goes is really important’ [BC-07]. A lack of understanding and appreciation of roles and mandates was reported both inter and intraprofessionally. A nurse with a background in PC and PH explained: ‘As a PHN, I encountered questions; I’d say [a] lack of support from other nurses who were in PC and other health care professionals who really didn’t understand what we did or were trying to do’ [Nat-02]. Many participants noted that negative impressions of the ‘other’ existed in both sectors. A physician with PC and PH experience explained: ‘There’s a feeling amongst a lot of PH officials, and bureaucrats and experts, etcetera that PC isn’t doing what it should be doing. […] It is partly a result, I believe, of misunderstanding each other’s roles and responsibilities’ [Nat-03]. Role clarity was supported when individuals had previous experience in both sectors.

Being flexible and adaptable in roles was another element of this factor. A PC program evaluator provided an example involving PC and PH nurses: ‘If you allow territories [or sectors] to evolve, but allow some crossover on those territories, so that if you’re not here today, I can actually pick up some of what it is that you do’ [Nat-08]. A pharmacist explained how flexibility in roles was related to expertise of staff:

So clear roles, [need to be] defined. And it doesn’t have to be the same in every location. Maybe a doctor feels comfortable doing diabetes education in one jurisdiction, where in another, it is more effectively delivered through nursing. So it doesn’t have to be rigid either. It should be fluid enough to allow for nuances and various expertise of people.

[NS-17]

With respect to jurisdictional differences, compared with ON and NS, BC, participants more frequently expressed having a lack of agreement on, and understanding of, roles and mandates than having agreement. NS participants talked more affirmatively about agreement on, and understanding of, roles and mandates than did ON and BC participants. BC participants expressed a lack of understanding and agreement about roles as influencing collaboration much more often than having understanding and agreement relative to ON and NS.

The above results regarding differences between provinces might be explained by BC’s primarily fee-for-service funded PC system in conjunction with system level changes that were occurring there around the time of the interviews. Changes included organizational restructuring within regional Health Authorities to redistribute PH functions and staffing, and restructuring of the physician workforce in PC (Population Health and Wellness: Ministry of Health, 2005; Wong *et al*., 2009). The integration of PH practitioners into non-PH departments where their roles and functions were not familiar to those already working there likely contributed to the lack of understanding.

As noted in our scoping literature review (Martin-Misener *et al*., 2012), others have noted that poorly understood roles and responsibilities, particularly across disciplines, significantly impedes PC and PH collaboration and other types of inter-organizational collaborations (D’Amour *et al*., 2008; Fuller *et al*., 2011). Clearly understood roles and responsibilities can enhance the efficiency and nature of decision-making among teams (Koelen *et al*., 2008; Sloane *et al*., 2009; Fuller *et al*., 2011), another influencing factor.

#### Effective Communication

Effective Communication had two elements: (a) *exchange of information* and (b) *facilitated engaged dialogue*. Exchange of information involved effective informal and formal sharing of information, ideas, and data related to patients/clients as well as educational and training activities. A PC nurse shared that they are, ‘trying to show that [exchanging information] it is a joint responsibility, and are just starting discussions with education’ [NS-04]. Other studies support our findings that these strategies ensure effective communication (Sloane *et al*., 2009; Schmied *et al*., 2010) and thus should be encouraged. Engaged facilitated dialogue, in the form of facilitated discussions between providers, supported communication efforts at an interpersonal level. One respiratory therapist said, ‘… something that needs to be there. You need facilitators to bring these groups together’ [ON-07]. Also, a lack of common language presented challenges for dialogue as this PC social worker explained:

The words are a problem. They have been and they continue to be. They are almost interchangeable but they mean different things to different people. That to me is a challenge around collaboration with PH or any other group.

[NS-14]

A PHN summed up the value of this factor this way: ‘…communication is the number one reason why collaboration will work or not work’ [BC-20].

Effective communication has been identified by others as being essential for effective collaboration. For example, a study of health care provider perspectives identified effective communication as a core competency required for interprofessional collaborative practice (Suter *et al.*, 2009). A more recent concept analysis of interprofessional collaboration related to chronic disease management found that attributes of interdisciplinary collaboration included effective and frequent communication which included shared documentation systems and regular interactions (Bookey‐Bassett *et al*., 2017). Our results support these findings and add new insights into specific strategies to operationalize effective communication in PC and PH collaborations such as introducing the role of facilitated discussions. Although facilitated discussions has been studied in the context of providers and patients (Lori *et al*., 2016) as well as in education (Dickey-Kurdziolek *et al*., 2010), more research is needed to study such techniques in PC and PH collaborations. The notion of thought-swapping could be potentially useful, in which participants must re-represent ideas presented by others (Dickey-Kurdziolek *et al.*, 2010).

#### Effective Decision Processes

Effective Decision Processes refers to clinical decision processes that involve the elements of (a) *effective practitioner problem-solving* and (b) *effective practitioner decision-making*. Effective practitioner problem-solving involves arriving at satisfactory responses to clinical problems or situations. A PC physician described practitioner problem-solving as ‘…problem solving and collaboration, that’s what we do everyday […] It’s trying to understand and listen to what people are doing, how it’s being done, how it can be done better. Where are we? Where are we missing things?’ [BC-18] Spending time to deeply understand shared problems and identify resources in the collaboration to address them is needed for effective problem solving. As noted by a NP:

I think being able to sit down and problem solve about population health issues […] on a very local level. And I really like to bring things down to the simple, to the neighborhood. What are some of the public health issues in the neighborhood and how can we as a team address them? And then have a plan and do the problem solving on this level and look at what resources we have within our neighborhood.

[BC-15]

Effective practitioner decision-making involves reaching satisfactory decisions in the collaboration. With respect to decision-making, a nurse noted that: ‘You need to begin to have shared decision-making’ [Nat-07]. Challenges were noted in collaborative decision-making as described by a physician: ‘I think in terms of decision making, I think it’s very challenging for docs who have been used to sort of coping on their own as solo cowboys. It’s very challenging to then understand team-based decision making’ [BC-07]. On the other hand, a nurse explained how effective decision-making can be enhanced: ‘We made decisions. We didn’t get hung up on little nibbling details and wordsmith things to death’ [NS-05]. Across provinces, participants from BC were more concerned about decision processes than ON or NS participants.

It is unclear why BC participants were more concerned about decision processes. The literature indicates that strong interprofessional collaborative practice can optimize clinical decision making across disciplines and organizations (Oelke *et al*., 2013; Bookey‐Bassett *et al*., 2017). Others have noted that a change in culture is needed in the socialization patterns of PC providers that supports ‘a willingness to share in patient care decision-making’ across disciplines with the inclusion of patients and their caregivers (Orchard *et al*., 2005). An interprofessional step-wise shared decision-making model developed by Légaré *et al*. (2011) can be a useful tool to support shared decision making in primary care and has potential to be applied to other health care systems, such as in PC and PH collaborations. More research is needed to explore this potential.

### Intrapersonal factors

#### Personal Qualities, Knowledge, and Skills

Personal Qualities, Knowledge and Skills includes three elements: (a) *experience or knowledge about collaboration*, (b) *leadership skills in collaboration*, and (c) *practitioner personal characteristics*. Many participants identified that experience or knowledge about collaboration facilitated collaboration; having experience in both PH and PC sectors was especially valued. One nurse explained:

And then of course [from PC] I went on to PH… And certainly brought my passion for medicine, surgery and maternal and child health with me into the field. And I really felt it was quite beneficial to my career in PH.

[NS-03]

Leadership skills in collaborations were described as being key. ‘It’s getting the right people and sharing or working according to your plan. And in some cases it’s quality of leadership’ [Nat-10]. Speaking about vulnerable populations, a PHN explained: ‘Seeing ourselves as change agents and people that actually can provide leadership and championship to build that same level of passion in folks [about people] that are probably in the most desperate of situations’ [NS-03]. Looking across provinces, participants from NS were more concerned than others about leadership skills. It is unclear why this was the case.

Closely linked to leadership was practitioner personal characteristics. A physician explained: ‘you need the right kind of personality that could bring these groups together’ [Nat-09]. Personal initiative was viewed as a valuable characteristic as this PC nurse practitioner explains: ‘She [colleague] just sort of made her own role evolve. She was a self-starter type of person. Built the program up, saw what needed to be done’ [ON-08]. A business administrator described personal characteristics that can also present challenges: ‘…it’s been more challenging in some clinics than others […] some of it is personality dependent’ [BC-11]. For example, an authoritarian style was identified as challenging. A business administrator explained: ‘They like to be in control. So if they have a nurse in their office, they’d like to hire the nurse rather than have somebody bumped into it because they have more union seniority or something like that’ [BC-01]. This quote also demonstrates the presence of a power dynamic that is present at the interpersonal level which is impacted by personal characteristics.

The factor, Personal Knowledge, Qualities, and Skills was supported by Koelen *et al*. (2008) who argued that intersectoral programmes tend to focus on achieving a goal rather than nurturing the process of collaboration. To address this requires leadership and a person with skills and qualities such as being flexible, reliable, visionary, and good at following up on decisions. Similarly, in an exploration of linkages among service providers in primary mental health care, Fuller *et al*. (2011) found that clinician attributes such as a commitment to collaboration, a flexible working style, and ability to fit into teams were important enablers for collaboration.

#### Personal Values, Beliefs, and Attitudes

Personal Values, Beliefs and Attitudes included two elements: (a) *willingness to collaborate* and (b) *responsiveness to patient, community, and provider needs*. Individuals who demonstrated willingness to collaborate enabled collaboration. A PH physician talked about the need to ‘be open to all the ideas, to be creative’ [BC-18] and demonstrate willingness to collaborate. Practitioners accustomed to working alone and unwilling to work within a team environment can present a significant barrier to PH and PC collaboration. With respect to responsiveness to patient, community, and provider needs, putting community and patient needs first was an enabler of collaboration. A PH physician argued that: ‘If you just respond to what people are telling you, you will be successful’ [BC-18]. Participants indicated it was also important to be sensitive to provider needs as described by this PHN who was setting up a project with an interprofessional PC team: ‘We come in […] being flexible to meet [PC’s] time constraints and meet their scheduling constraints’ [ON-18].

The element willingness to collaborate is consistent with findings from a Q-methodology study that explored common key informant viewpoints (Akhtar-Danesh *et al*., 2013) that reflected personal values, attitudes, and beliefs about PC and PH collaboration. Three distinct viewpoints included people who were: ‘system driven collaborators’; ‘cautious collaborators’; or ‘competent isolationists.’ ‘Competent isolationists’ were not interested or willing to collaborate (Akhtar-Danesh *et al*., 2013). Identifying those who may be more willing (ie, ‘cautious collaborators’ and ‘system driven collaborators’) may be advisable when initiating a collaboration. Furthermore, professional education and development programs should include content on processes to strengthen collaboration among sectors rather than focusing on interprofessional collaboration alone. This can help to encourage the formation of positive knowledge, beliefs, and attitudes around the benefits of collaboration.

Responsiveness to community need is particularly important for PC and PH collaborations aimed at supporting effective community-based programs and services. This is rooted in the history of Lilian Wald’s contribution to the development of public health nursing practice in which PHNs are expected to ‘invent a diverse mix of public and private programs that respect local custom, link effectively with mainstream health care institutions, and are substitutive, additive, or complementary to community needs’ (Buhler-Wilkerson, 1993: 1784). This is also embedded within components of the model of community-oriented PC which includes working with a defined population and ‘a process to address the health problems of a community’ (Nevin, 2005).

### Relationships among factors

**Figure 1:**
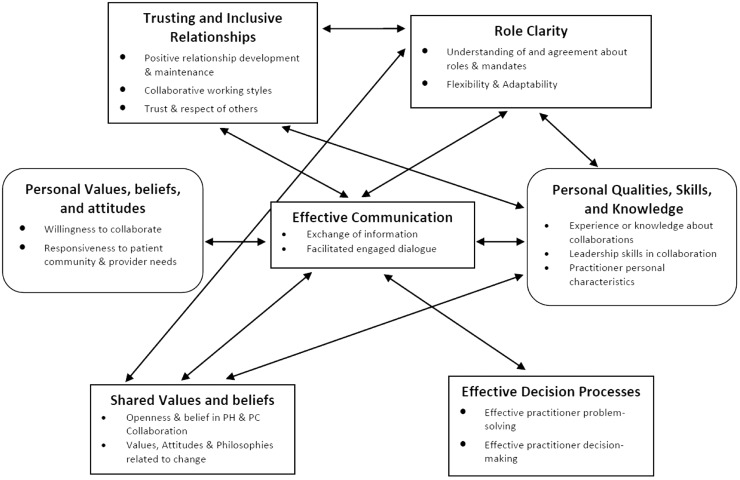
Commonly reported relationships among influencing factors for primary care and public health collaboration. Note: Interpersonal factors (rectangle); intrapersonal factors (rounded rectangle)

We identified instances where participants spoke about interactions among most factors at the interpersonal and intrapersonal levels indicating that they do not operate independently. Here we highlight the most common relationships among intra and interpersonal factors to illustrate the complexity of these interactions (Figure 1).

The factor –Trusting and Inclusive Relationships –was most often associated with Effective Communication and Role Clarity. A PHN explained how the value of trusting relationships was understood as a key to understanding roles and enhanced effective communication.

I think there’s some real value in developing [PC and PH] relationships between staff. That helps with understanding roles, and increases communication. If you’re actually working face-to-face with someone, and then you call them, they’re more apt to pick up the phone.

[ON-19]

A PC business administrator reflects on the relationship between the factors Trusting and Inclusive Relationships and Role Clarity, which then leads to an increase in Shared Values Beliefs and Attitudes by seeing the benefits of working in collaboration.

Basically it comes down to trusting each other and understanding the limits and the scope of each other’s roles and disciplines. Then gradually that trust is built and they recognize that, if they share the care together, they can actually see more patients and provide better care in the long run.

[BC-11]

There were also interactions found between inter and intrapersonal factors. Interpersonal factors –Trusting and Inclusive Relationships, Role Clarity, Effective Communication, and Shared Values Beliefs and Attitudes (being responsive to the needs of the community) interacted with an intrapersonal factor Personal Qualities, Knowledge, and Skills. The following quote from a PHN demonstrates this complex interaction.

The nurse practitioner and I collaborate together all the time if there is something that has been asked of either of us in the community. From the time [the nurse practitioner] started… The first thing she did was visit every single person that was working in the area- every service provider- everyone, and found out what their role was, how she could work with them to make things work. So again, laying that foundation [of trust].

[NS-08]

Numerous relationships between and among factors were evident throughout the data confirming the complexity of interactions at the intra and interpersonal level.

These results provide a deeper understanding of intra and interpersonal factors influencing collaboration and the relationships between them. At the interpersonal level individual health professionals engage in practice with each other. Forming Trusting and Inclusive Relationships between individuals working in PH and PC is foundational for Effective Communication, Decision-Making Processes, Shared Values, Beliefs and Attitudes and Role Clarity. These factors enable individuals to co-construct ways of working together, in which each person can use their health sector expertise to collaborate toward shared goals. The relationship between Trusting and Inclusive Relationships and the intrapersonal factor Personal Qualities, Knowledge and Skills has been explained by others. Covey and Merrill (2006) identified two essential components necessary to build trusting relationships that are related to intrapersonal characteristics, namely character and competence. Character involves personal integrity, motivation, and intent. Competence includes capabilities, skills, results, and a track record. Both are vital intrapersonal characteristics that are needed to build trusting relationships and in turn successful collaborations.

## Discussion

Our results expand on inter-organizational collaboration models developed by D’Amour *et al*. (2008) and San Martín-Rodríguez *et al*. (2005). We highlight the interactional dimension and explore interpersonal factors in-depth. We also examine intrapersonal factors that are largely ignored by others in relation to their influence on successful collaboration between PH and PC. Interpersonal factors necessary for successful collaboration between PC and PH were: Trusting and Inclusive Relationships; Role Clarity; Effective Communication; Shared Values, Beliefs, and Attitudes; and Effective Decision Processes. At the intrapersonal level the factors were: Personal Qualities, Knowledge, and Skills and Personal Values, Beliefs and Attitudes.

Among the three provinces, there was minimal inter-provincial variation and perspectives about influencing factors and their elements were similar across sectors reflecting a general convergence of opinion of what makes PC and PH collaboration work or not. BC participants were more concerned about decision-processes and had a lack of agreement on and understanding of roles and mandates, while NS participants were more concerned about lack of leadership skills for collaboration compared with the other provinces. However, overall, interprovincial differences were generally minimal. This might be explained by the fact that human characteristics in collaborations are not as likely to vary across provinces or sectors in the same way that organizational, systemic or collaboration structural influences might. More importantly, the results suggest there is a complex interplay of the relationships among factors that is based on power relations between professions (physicians and nurses) as well as PC and PH sectors. Power, or the ability or capacity to act or exercise influence (Lukes, 1974), was discussed by participants in terms of the lack of effective communication and a lack of knowledge about different health professions’ roles, mandates, or scope of practice. Past work suggests that professional groups aim to secure and protect exclusive areas of knowledge by regulating entry and work practices to gain economic, social, and political advantage (Freidson, 1988; Reeves *et al*., 2010). The inherent and often unspoken power relations make collaboration across health professionals and sectors especially challenging.

A lack of Role Clarity coupled with a few opportunities for Effective Communication or reflective practice can contribute to boundary tensions (Booth and Hewison, 2002; Reeves *et al*., 2010), conflict (Baker *et al*., 2011), and undermining of individuals’ ability to collaborate effectively (Fox and Reeves, 2015). Further, work by Baker *et al*. (2011) reveals that attitudes and beliefs about the value that each profession places on their own knowledge and skills could translate into a lack of knowledge of roles and responsibilities of their colleagues, that is, role clarity, particularly across sectors. Providers and their managers are encouraged to revisit their roles often within teams as they may need to alter them should the needs of the collaboration change over time or new providers join the collaboration. It may also be advantageous to put roles in writing so that they are well understood and more easily shared and communicated.

Many inter and intrapersonal factors identified in this research support the literature on relational practice. Relational practice refers to ‘the wide range of off-line, backstage, or collaborative work that people do which goes largely unrecognized and unrewarded in the workplace.’ The traditional view of relational practice as ‘women’s work’ was rightly questioned by Holmes and Marra (2004: 377). Relational practice activities, which occur at the boundaries of meetings, such as discussions in corridors or over lunch traditionally have not been considered ‘real work.’ However, Holmes and Marra argue that these activities are critically important because relational work supports working together. Hartick-Doane (2014) argues that:

Relationships are often discussed and understood as the “soft” part of nursing – the touchy, feely stuff that one does when one has time. This understanding stands in stark contrast to the rich history of scholarship in nursing that illuminates how nursing is a skillful relational process.

This highlights the essential value of building relational practice skills for all disciplines, which is particularly relevant for those involved in PC and PH collaborations, given the inter and intrapersonal influencing factors that have emerged from this research.

As far as limitations, although our interviews included participants primarily from three provincial health care contexts, greater representation in our sample from diverse populations and other Canadian provinces and territories including First Nations communities would have been desirable had resources permitted.

## Conclusion

This descriptive interpretive study explored perceptions of 74 Canadian key informants who were working in or knowledgeable about PC and PH collaboration. It built on a scoping review of the literature to identify factors that influence PC and PH collaboration in relation to the nature of collaborations that exist in Canada. This paper focuses specifically on inter and intrapersonal factors influencing successful collaboration. It identified five interpersonal and two intrapersonal factors. While there were very few inter-provincial or intersectoral differences found, most factors interacted with each other, thereby highlighting the complex and interconnected nature of this work. What is unique here is the exposure of particular factors and their elements that are particularly relevant to PC and PH collaboration, such as having knowledge of and experience in the other sector and being responsive to patient, community, and provider needs. Interprofessional practice team members and administrators working across PC and PH sectors as well as educators need to attend to the development and support of interpersonal interactions and intrapersonal qualities and skills that can impact on collaboration. This paper provides a practical place to start.
